# Population Dynamics of the Rubber Plantation Litter Beetle *Luprops tristis*, in Relation to Annual Cycle of Foliage Phenology of Its Host, the Para Rubber Tree, *Hevea brasiliensis*

**DOI:** 10.1673/031.009.5601

**Published:** 2009-07-15

**Authors:** Thomas K. Sabu, K.V. Vinod

**Affiliations:** Litter Entomology Research Unit, Post Graduate and Research Department of Zoology, St. Joseph's College, Devagiri, Calicut, Kerala, India 673 008

**Keywords:** Mupli beetles, aggregation, *Hevea brasiliensis* RRII 105

## Abstract

The population dynamics of the rubber plantation litter beetle, *Luprops tristis* Fabricius 1801 (Coleoptera: Tenebrionidae) was assessed in relation to the phenology of leaf shedding and defoliation pattern of para rubber trees, *Hevea brasiliensis* Müll. Arg (Malpighiales: Euphorbiaceae), during a two year study period. The abundance of adults, larvae and pupae per 1m^2^ of litter sample was recorded. Post dormancy beetles appeared in leaf litter following annual leaf shedding, whereas larvae, pupae and teneral adults were present after leaf flush. No stages were recorded from plantations following the summer rains until the annual litter fall in the next season. Parental adults peaked at the time of leaf sprouting and tender leaf fall. Larvae and teneral adults peaked at the time of premature fall of green leaves and flowers. Teneral adults of six age classes were recorded and all entered dormancy irrespective of the feeding time available to each age class. Females outnumbered males in the parent generation, while the sex ratio of new generation adults was not biased towards either sex. The phenological stages of rubber trees included leaf fall in late December and early January, leaf sprouting and new leaf production in January and flowering in February. All feeding stages of *L. tristis* peaked in abundance when premature leaves are most abundant in the leaf litter. Prediction of the timing of appearance of various developmental stages of *L. tristis* in plantations, invasion into buildings and intensity of population build up in rubber belts is possible by tracking the phenology of leaf fall in rubber plantations, time of return of post dormancy adults and the onset of summer rainfall. Perfect synchrony was recorded between the field return of parental adults with annual leaf shedding, the oviposition phase of parental adults with tender leaf fall at the time of leaf sprouting, and larval and teneral adult stages with premature fall of leaves. Premature leaf availability is suggested as contributing to the reproductive efficiency of parental adults, the survival of early developmental stages and of new generation adults during dormancy.

## Introduction

Massive seasonal invasion of huge aggregations of litter dwelling darkling beetles *Luprops tristis* Fabricius 1801 (Coleoptera: Tenebrionidae), following summer showers and their prolonged stay in a state of dormancy (oligopause) is a seasonal nuisance for farming communities in the rubber plantation tracts of Kerala along the western slopes of the Western Ghats (Sabu et al. 2007, 2008). Clusters of several thousands of invading beetles crawl inside living rooms and often fall off from ceilings. Subsequently they congregate in dark, undisturbed areas such as attics and wall voids and remain dormant for nine months. They do not sting or bite, however, when disturbed (such as picking them off the walls or when they are squashed or pressed against while sleeping) they release an irritating odoriferous phenolic secretion that causes burns on the skin. With their detritivorous habits, harmless nature, nocturnal surface activity and diurnal passivity in lower litter layers, they would have remained inconspicuous facilitators in litter decomposition and nutrient cycling in monoculture “rubber forests” in the region. However, their massive seasonal invasion into traditional tile roofed residential buildings and thatched sheds make them the most dreaded beetles in rubber-growing regions.

*L. tristis* beetles breed in the litter stands of rubber plantations and all feeding stages have a strong preference towards tender leaves. Larval, pupal and teneral adult stages were observed in the field only after annual leaf fall and biology studies indicated that their life cycle is linked to the phenology of leaf shedding of rubber plants (Sabu et al. 2008; Joby 2006). Understanding the relationship between the phenology of leaf shedding of rubber plants and *L. tristis* is central to understanding its life history and population dynamics. Population management depends on accurate forecasting of seasonal activity and insect phenology, which deals with the timing of recurring biological events in relation to key environmental factors, is fundamental to understanding population growth and insect migration behaviour. Knowledge of the phenology of host and pest beetles may allow prediction of the timing of appearance of particular developmental stages in the field and requirements for life stage development, that may have potential for management (Nahrung and Allen 2004; Tauber and Tauber 1976). Here we present the results of two years of field monitoring of life stages, abundance patterns and timing of invasion and how the leaf phenology of rubber and the population dynamics of different stages of *L. tristis* are related.

## Methodology

### Study site

**Figure 1.**
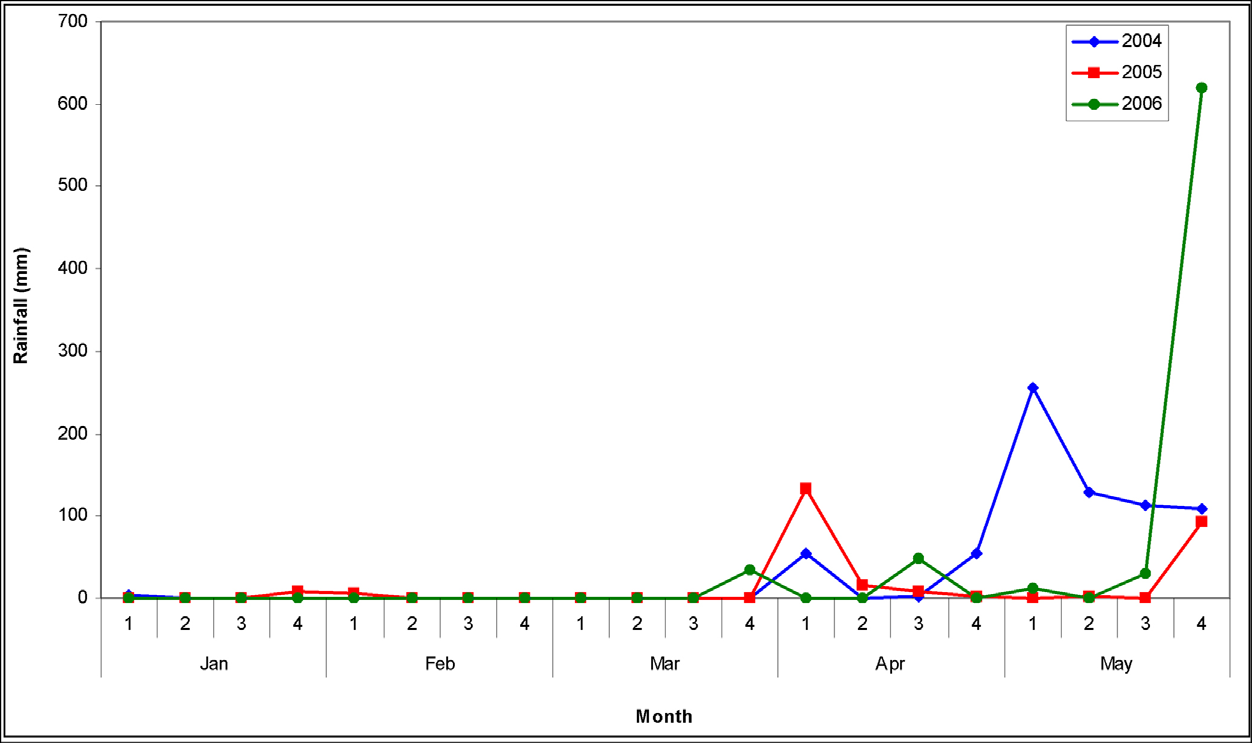
Rainfall data recorded during 2004–2006 study period at Thamarassery (All values in mm).

Studies on the population dynamics of the beetle and foliage phenology of rubber tree were carried out in the middle of a 10 year old, 20 hectare plantation of para rubber trees, *Hevea brasiliensis* Muell. Arg. clone RRII 105, (Malpighiales: Euphorbiaceae) with history of *L. tristis* incidence. The study site is located 30 km east off the Malabar Coast at Thamarassery (11° 26′ N, 75° 53′ E), in the Kerala state of India. Thatched residential buildings with regular occurrence of beetle invasion were present in the vicinity of the plantation. Southwest and the northeast monsoons control the climate of the region. Average rainfall for two consecutive years (2005–06) in the study habitat was 3546.8 mm. Occasional summer showers occur in March, April and May (Figure 1). Temperature varies annually between 23–32°C. Relative humidity is in the range of 68–93% (CWRDM 2006).

### Host plant

*H. brasiliensis* is a deciduous perennial tree with a major annual leaf shedding during December, leaf flush in January and flowering in February. It is a native of the rain forests of Amazone lying 5° south and north of Equator. Among the 10 species of the genus, *H. brasiliensis* is the only species grown commercially as source of natural rubber. It produces about 99% of the natural rubber consumed (George and Panicker 2000; Premakumari and Saraswathyamma 2000). Rubber is a constituent of latex produced in the lactiferous tissue of the plant. Commercial rubber plantations of about half a million hectares are present along the western slopes of the Western Ghats in the south Indian state of Kerala (ER 2006) and 85% of the rubber production in India takes place here. RRII 105 is the most widely cultivated clone in the region.

### Study organism

*L. tristis,* earlier referred to as *L. curticollis* (regionally known as *Mupli* beetle) is a detritivorous, litter-dwelling univoltine beetle (Sabu et al. 2007). It is a prominent fraction of rubber litter insect fauna during the dry months Joby 2006; Vineesh 2007) and enters a nine month long dormancy in residential buildings prior to the onset of monsoon season. Its life cycle involves a five instar larval phase, a short duration pupal phase and an active adult phase in rubber litter.

### Sampling

The chronology of leaf phenology including leaf shedding, refoliation involving sprouting and new leaf flush, maturation, premature leaf fall, flowering and flower fall of 625 plants in a 100 m × 100 m plot were monitored weekly from December to May of 2004–05 and 2005–06. The percentage of trees in each phenology class was recorded each week.

Activities of aggregated, dormant beetles in thatched buildings in the vicinity of the study plot were observed on a weekly basis following post rainy invasion of beetles in April 2004 and April 2005. Earlier studies (Sabu et al. 2008) showed that *L. tristis* returns to rubber litter following annual leaf fall in rubber plantations in the second week of December. Abundance of *L. tristis* beetles in plantations following their return was checked for six months from the first week of December to the last week of May in 2004–05 and 2005–06.

Leaf litter present inside a 1 m^2^ area was collected by placing a 1 m^2^ wooden quadrat frame onto the litter and then scraping up all litter and loose humus from within the frame area into a large polyethylene bag. Litter collected for analysis refers to the upper organic litter plus the loose humus layer. Samples for the extraction were kept undisturbed in the polythene bags, individual beetle stages were extracted employing Winkler sifting methods (Besuchet et al. 1987). Each sample was sifted through a wire sieve with square holes of 1 cm × 1 cm into a 2 m^2^ white plastic sheet. Adults and larvae were hand picked in the order of fast moving larvae and adults followed by immobile pupae and dead adults and transferred to 70% alcohol in labelled containers. Abundance of each stage (identified as larvae, pupae, teneral adults and post dormancy adults) per 1m^2^ of litter sample was recorded. Individual larval instars were not considered due to the small size and short duration of 1^st^ and 2^nd^ larval instars and the absence of external features to distinguish the stages. Larvae were categorized into stage 1 (< 6mm) and stage II (≥ 6mm) based on size. All adults collected up to the field presence of pupae were categorized as parental adults and those present afterwards were categorized into parental and teneral adults based on rigidity of elytra, which was determined by compressing the elytra laterally between a pair of forceps (Nahrung and Allen 2004). Sexing of adults was done following the sternal notch methodology (Vinod et al. 2008). Field appearance of parental adults was recorded in relation to annual leaf shedding and of larvae, pupae and teneral adults in relation to new leaf flush. Thirty samples of litter were collected per week at random from the plantation, maintaining sampling intervals of 10 m between samples. Time of sampling was between 18:30 h and 20:00 h when the beetles were active in field. Diurnal sampling was not done, as beetles were less obvious in the field as they were hiding behind leaf litter and stones.

### Statistical analysis

Significance levels of variation in weekly abundance of each life stage in both the years of study was analysed with two way ANOVA. Megastat, version 10.0 (Orris 2005) was used for all statistical analysis.

## Results

### Phenology of rubber trees

**Figure 2.**
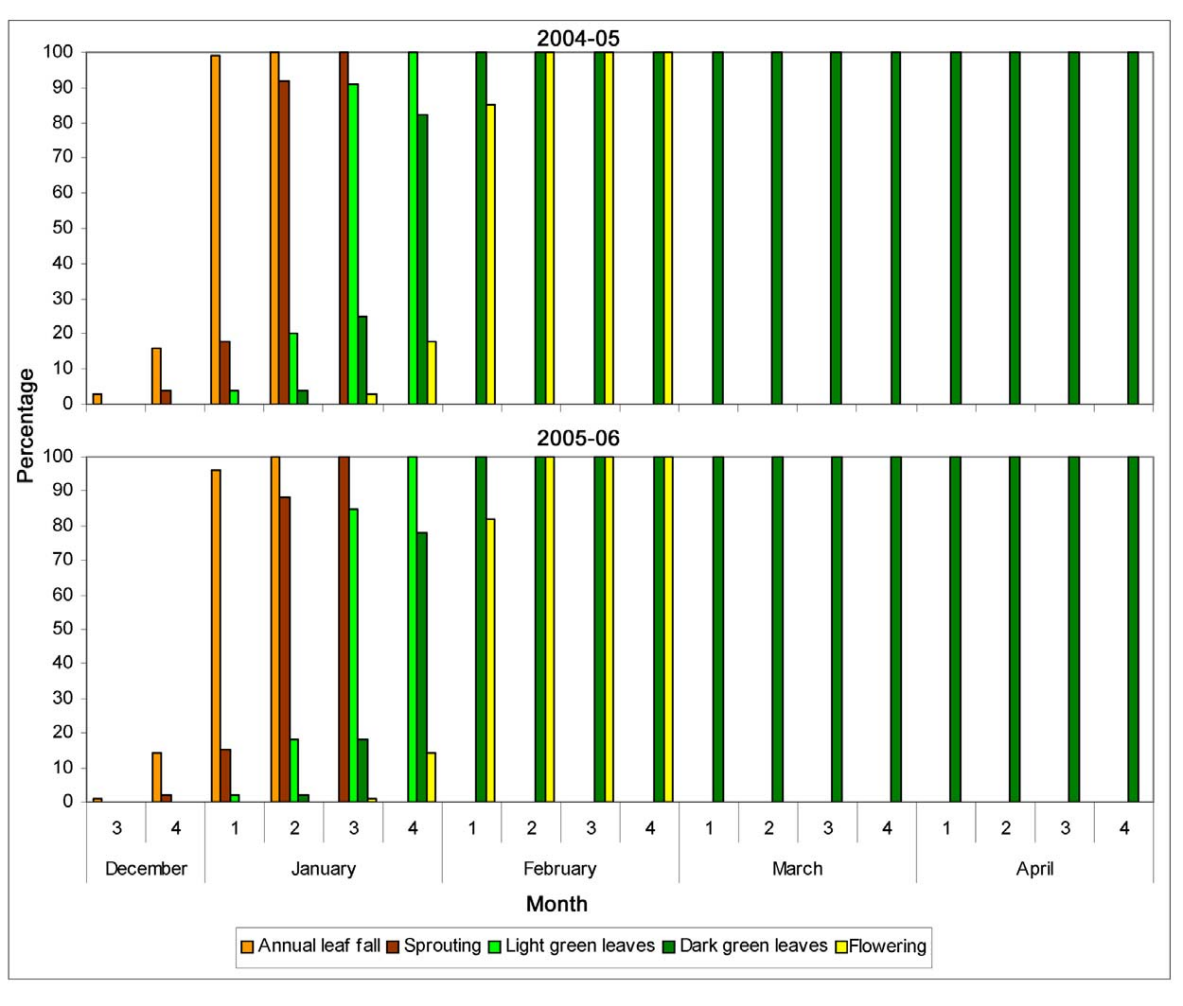
Phenology of leaf fall, leaf flush and flowering in a rubber plantation at Thamarassery during 2004–2006 study period. Numbers on the X axis are weeks. Values on the Y axis in percentage.

**Figure 4.**
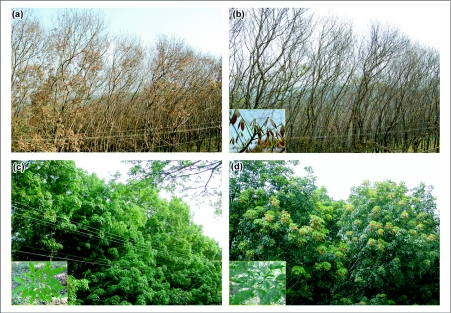
Different stages of leaf phenology in RRII 105 plantation, a) Annual leaf fall, b) New leaf sprouting and tender brown leaf, c) Green leaf formation and powdery mildew affected light green leaf and d) Dark green leaf and flowering stage and powdery mildew affected dark green leaf. Inset images shows 1. Tender brown leaves, 2. Powdery mildew affected light green leaves and 3. Powdery mildew affected dark green leaves.

A broad classification of the phenology of RRII 105 clone of rubber trees in the region (Figures 2, 3, 4) is as follows.

### Leaf fall

Most trees (82% in 2004–2005 and 78% in 2005–2006) started litter fall in 2^nd^ week of December. Three percent of the trees in 2004–05 and 1% in 2005–06 started leaf shedding one week earlier (1^st^ week of December), 15% in 2004–2005 and 21% in 2005–06 started one week later (3^rd^ week of December). Sixteen per cent of the trees completed leaf shedding in the 4 week of December in 2004–05 and 14% in 2005–06. More than 95% of the trees completed leaf shedding in the 1^st^ week of January itself (99% in 2005 and 96% in 2006). The remaining trees (1% in 2005 and 4% in 2006) completed leaf shedding by the 2^nd^ week of January (Figures 2, 3).

### Leaf Sprouting

Sprouting started in December 4 week (4% in 2005 and 2% in 2006). Spouting of 92% of the trees was completed in the 2^nd^ week of January in 2005 and 88% in 2006. Regular fall of brown tender leaf by wind mediated snapping and collision of branches was noticed during the period of sprouting.

### Light green leaves

Tender brown coloured leaves turned light green from the 1^st^ week of January onwards (4% in 2005 and 2% in 2006). By the 3^rd^ week of January, more than 80% of the trees became light green (91% in 2005 and 85% in 2006). The presence of powdery mildew was correlated with premature fall of light green leaves.

### Flowering

Flowering started in the 3^rd^ week of January (3% in 2005 and 1% in 2006). Eighty five percent of the trees were flowering in 2005 and 82% in 2006 three weeks following leaf sprouting (1^st^ week of February). The whole plantation was flowering by 4 weeks following leaf sprouting (2^nd^ week of February). Flowers were present until the 4 week of February in 2005 and 2006. Flower fall was observed during the entire flowering period (3^rd^ week of January to the 4 week of February) and peaked 5 weeks following leaf sprouting in the 3^rd^ week of February.

### Dark green leaves

Light green leaves of 4% of the trees turned dark green in the 2^nd^ week of January in 2005 and 2% in 2007. Light green leaves of more than 75 % of the trees turned dark green, two weeks following sprouting (4 week of January), (82% in 2005 and 78 % in 2006), and entire trees turned dark green, three weeks following sprouting (1^st^ week of February). Powdery mildew mediated premature leaf fall was recorded throughout the flowering stage and the highest level of premature fall was observed 8 weeks following new leaf formation (2^nd^ week of March) in both study periods.

**Fleure 3.**
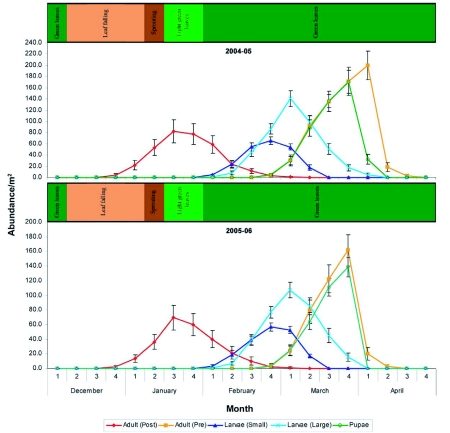
Population dynamics (mean ±SD) of each life stage (larvae (small), larvae (large), pupae, adult (post) and adult (pre)) collected per m^2^ of litter in relation to the phenology of leaf fall and leaf flush in a rubber plantation at Thamarassery during 2004–2006 study period.

### Population biology of *L. tristis*

From April to December, beetles were dormant and remained aggregated without any physical activities within shelters. No beetles were recorded in the litter of the plantation during the period. Arousal from dormancy and attraction towards light and nocturnal activity (refractory phase) was noticed in the 4 week of December and lasted for three weeks (15–17 days).

Overwintered parental beetles were recorded in the leaf litter two weeks following the beginning of annual leaf fall (4^th^ week of December to the 1^st^ week of March) (Figure 3). Abundance of beetles gradually increased and peaked 2 weeks following complete leaf fall and one week following new leaf formation (3^rd^ week of January) and declined 3 weeks following leaf fall (4^th^ week of January). Significant variation in weekly abundance was observed between as well as within sampling years (between years df = 1, F = 88.53, P <0.05; among weeks df = 9, F = 426.6, P <0.05). Parental adults were not recorded beyond 10 weeks following their field return (March 2^nd^ week). Dead parental adults were recorded in sifted samples 4 weeks after field return (4^th^ week of January) and their numbers peaked 5–6 weeks following return to plantations (2^nd^ week of February in 2005 and 1^st^ week of February in 2006). During both study periods (2005 and 2006), parental adults returned to the field two weeks following the beginning of annual leaf fall, peaked and declined during the same period (Figure 3). The sex ratio of post dormancy adults was 1:1.3 (male:female).

### Larval instars

Larval stages I and II appeared three weeks (1^st^ week of February) following new leaf formation. Significant weekly variation in the abundance of larval stages was observed (between years df = 1, F = 94.59, P <0.05; among weeks df = 5, F = 1064.57, P <0.05 and between years df = 1, F = 179.95, P <0.05; among weeks df = 8, F = 2095.31, P <0.05 for Larva I and Larva II respectively). Larva I peaked 6 weeks (4^th^ week of February) and Larva II peaked 7 weeks (March 1^st^ week) following new leaf formation when leaves were dark green in color and powdery mildew mediated leaf fall peaked. Abundance of Larva I and II declined 7 and 8 weeks respectively, following new leaf formation (1^st^ and 2^nd^ weeks of March respectively). Larval stage I was recorded up to 8 weeks following new leaf formation (2^nd^ week of March) and Larval stage II until arrival of first summer showers: 11 weeks in 2005 (1^st^ week of April) and 10 weeks in 2006 (4^th^ week of March) following new leaf formation.

### Pupae

**Figure 5.**
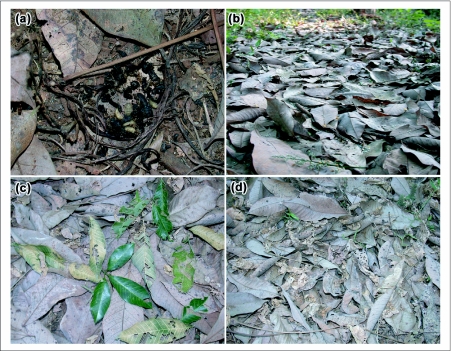
Leaf litter of rubber plantation during the leaf sprouting and flowering phase, a) Lower leaf litter layers with late stages of Vth instar larvae, larval exuviate and pupae, b) & c) *Luprops trisits* fed light green leaves and untouched dark green leaves in leaf litter and d) leaf litter with fallen flowers.

Pupae first appeared six weeks after new leaf formation (4^th^ week of February), when leaves were dark green in color and peaked during the 10 week following new leaf formation (4 week of March), when leaves were dark green (Figure 5). Significant weekly variation in the abundance of pupae was observed (between years df = 1, F = 317.56, P <0.05; among weeks df = 5, F = 1985.94, P <0.05). No pupae were recorded after first summer showers, 12 weeks after new leaf formation in 2005 and 11 weeks after new leaf formation in 2006 (2^nd^ week of April in 2005 and 1^st^ week of April in 2006).

### Teneral adults

Teneral adults appeared six weeks following new leaf formation (4^th^ week of February) in both the years when the plantations were in light green leaves. Their abundance peaked 11 weeks following new leaf formation (1^st^ week of April) in 2005 and 10 weeks following new leaf formation (4 week of March) in 2006. Two way ANOVA results showed significant variation in weekly abundance of teneral adults (between years df = 1, F = 520.24, P <0.05; among weeks df = 7, F = 1170.28, P <0.05). Their abundance declined drastically during 1^st^ week of April in 2005 and 4^th^ week of March in 2006 after the onset of summer showers. No beetles were recorded two weeks following summer shower (4^th^ week of April in 2005 and 3^rd^ week of April in 2006). Sex ratio of teneral adults was 1:1.08 (male and female).

### Overlap in adult generations

The simultaneous presence of parent and teneral adults was recorded for two weeks (4^th^ week of February in 2005 and 1^st^ week of March in 2006), 7–8 weeks following complete leaf fall.

## Discussion

This study reveals that by tracking the phenology of leaf fall in rubber plantations, timing of return of post dormancy adults of *L. tristis* and relating these with the onset of summer rainfall enables the prediction of the seasonal appearance of subsequent developmental stages of *L. tristis* in plantations, the timing of their invasion into buildings and the intensity of population build up in rubber plantations. The stages of rubber plant growth, with sprouting, new leaf production, flowering and leaf fall are spread over four months. Feeding stages of *L. tristis* peak in abundance when the resource (premature leaves) they exploit is most abundant. The data show the coincidence of the return of parental adults from buildings to the field with annual leaf shedding and the oviposition phase of parental adults with leaf sprouting. Tender leaf fall and prolonged premature leaf fall during the presence of larval and teneral adult stages in leaf litter must enhance the reproductive efficiency of parental adults, survival of early developmental stages and of new generation adults during dormancy. Hence, timing of both annual and premature leaf fall, with the appearance and duration of developmental life stages in leaf litter strongly influences the population dynamics of *L. tristis.*

The termination of dormancy and return to the field of post dormancy adults perfectly coincided with the end of annual leaf fall and fall of sprouting leaves in rubber plantations. It appears that rubber trees and dormant beetles may respond to the same environmental cue; rubber trees shed leaves well in advance of the onset of the dry season as a mechanism to prevent water loss (Santiago et al. 2004; Santiago and Mulkey 2005) and the return of dry conditions possibly leads to the arousal of *L. tristis* from dormancy (Sabu et al. 2008). This synchrony of termination of dormancy and subsequent field return of beetles with annual leaf fall and availability of prematurely fallen tender leaves is suggested as significantly contributing to the unprecedented abundance of *L. tristis* which is unheard of in natural or monoculture litter stands. It appears that *L. tristis* synchronises dormancy termination with annual leaf shedding and new leaf flush, which occur concurrently in plantations. Further, dormancy during the six month monsoon period, when litter habitat is less conducive for beetles, being wet and in the advanced phases of faster decomposition Joby 2006), allows synchronization of the *L. tristis* life cycle. It enables synchronized development of post dormancy adults irrespective of age class variations in the following dry season when the leaf litter habitat becomes suitable by the influx of fresh litter and the availability of tender leaves as a food resource. The simultaneous return of post dormancy adults to the litter habitat facilitates synchronized feeding and preparation for breeding activities through temporal and spatial coincidence of mates. Dormancy in *L. tristis* becomes a classical example of how seasonal patterns of development optimize reproductive efficiency by synchronising mating (Denlinger 1986) and food availability. Synchronized development of feeding stages with fresh fallen leaves enables efficient utilization of the tender leaf resources that are transient in nature, and the brief period of dry litter habitat provide conditions for larval, pupal and teneral adults before the onset of the long wet season.

Premature leaf fall occurs at two stages, the first at the time of leaf sprouting coinciding with the return of fooddeprived post dormancy adults and, later when larvae and teneral adults are abundant in leaf litter, ensuring that all feeding stages are in receipt of a continuous supply of preferred leaf resources. Furthermore, fallen flowers are another resource available to the beetles for a brief period of 5 weeks. Based on the strong preference of all feeding stages towards premature leaves (Sabu 2007; Sabu and Vinod in press), we suggest that control of powdery mildew-mediated premature leaf fall Jacob 2002) will limit the availability of tender leaves to larval stages and teneral adults. Implications of the limited availability of tender leaves to various developmental stages need to be experimentally ascertained to understand whether control of powdery mildew disease mediated premature leaf fall would enable regulation of the population build up of *L. tristis* beetles in rubber belts.

The presence of dead parental adults in litter samples, even when prematurely fallen leaves are readily available and a significant proportion of the larval population exists in the field, indicate that parental adults reached the end of their nine month long life span (Sabu et al. 2008). This observation substantiates the earlier findings that adults are univoltine and the longevity of *L. tristis* ends 5–7 weeks following their return to the field from dormancy (Sabu et al. 2008). The absence of dead larvae and teneral adults at any stage in leaf litter indicates that shortage of food resources is not affecting the population build up of the new generation and also their ability to survive in rubber leaf litter. Further, the decline of parental adults timed with the arrival of teneral adults and larvae avoids significant overlap of generations and competition for the preferred tender leaves.

The increase in the number of dead post dormancy adults in litter samples, four weeks following their return to the field, and one week prior to the emergence of larval instars, raises an important question about the relationship between the phenology of litter fall of different *Hevea* clones and habitat and feeding requirements of post dormancy adults. Among rubber clones cultivated in the region, the annual leaf shedding of RRII 105 alone coincides perfectly with the field return of *L. tristis;* for other clones leaf shedding starts two weeks later Joby 2006). Consequently, we speculate that in the absence of RRII 105 clones, post dormancy beetles would face a two-week delay in suitable litter habitat with which to start breeding activities. In the absence of RRII 105 plantations, delayed leaf fall by 2–3 weeks in other clones may lead to non-availability of suitable litter habitat and preferred tender leaves to post dormancy adults and consequently high early mortality of post dormancy adults and lesser reproductive efficiency of the surviving beetles. Currently, mating pairs are observed in the first week after the return of post dormancy adults, and eggs and first instar larvae appear in the second week after return, leading to the formation of a subset of teneral adults with a very long period of feeding time prior to their entry into dormancy. Dead parental adults were observed for a six week period (4^th^ week of January to 1^st^ week of March) even in the presence of readily available tender leaves at the time of their return to the leaf litter. Consequently, a 2–3 week (4 week of May) delay in getting a litter habitat and tender leaves is certain to affect the feeding and reproductive efficiency of post-dormancy beetles and the active feeding time available to teneral adults. It raises the question of employing rubber clones with delayed leaf falling pattern or utilizing the intraclonal variation in leaf shedding of RRII 105 clones as a cost effective control measure to reduce population build up of *L. tristis.* This study predicts that introduction of rubber clones whose leaf shedding does not coincide with post dormancy return of *L. tristis* and sheds litter later than RRII 105 clones will lead to low population build up of *L. tristis,* and the introduction of clones that shed leaves earlier to the post dormancy return of *L. tristis* will lead to rise in population build up of *L. tristis.* Two new clones, RRII 430 and RRII 414, introduced as replacements for the widely cultivated RRII 105, shed leaves 15 days later than RRII 105. How they affect the population build up and life cycle of *L. tristis* needs to be analyzed. Since RRII 105 is the most widely cultivated clone (Saraswathyamma et al. 2000) and young plantations of various ages are present in the region, RRII 105 plantations will provide a suitable habitat for *L. tristis* for another 10–15 years and switching over to new clones and the compete shutting out of the availability of timely litter habitat and tender leaves will be a gradual process. That leaves the control of premature leaf fall in the existing rubber plantations and limiting the availability of preferred food resources as a more practical option for the control of *L. tristis,* which needs to be experimentally tested.

The emergence pattern of teneral adults shows that there are six age-classes of teneral adults with those emerging in the 1^st^ week getting 6 weeks of feeding time, 2^nd^ week arrivals getting 5 weeks etc., whereas those in the last week before the onset of summer rains get hardly one week. The highest abundance of tenerals was recorded in the 1^st^ week of April in 2005 (five weeks after appearance in the field) and the 4^th^ week of March in 2006 (four weeks after appearance in the field). The highest formation of tenerals occurred two weeks following their appearance in both years (2^nd^ week of March) with a rise of 30.8% and 34.1 % in 2005 and 2006 respectively. Since the age class of tenerals varies based on their emergence date, those with a longer age class have more feeding time and hence more reserve food to tide over dormancy than the late developed short age class adults (Sabu et al. 2008). This supports the earlier findings that the duration of teneral adults before dormancy, the active feeding time available to them and the timing of onset of summer showers are the important factors deciding the population build up of *L. tristis.* Since field return of post dormancy adults, summer rainfall and home invasion of teneral adults occurred in the same period in 2003–04 Joby 2006), 2004–05, and 2005–06 (present study) periods, in the absence of empirical data we speculate that variation in the availability of prematurely fallen tender leaves might have lead to the differences in field abundance of beetles between years and not variation in the incidence of rainfall.

Analysis of the amount of fat body required to tide over the nine month period of dormancy and feeding duration required for sufficient build up of fat body needs to be measured to predict the effect of variable duration of feeding time in the life span of *L. tristis.* Our observations in the rubber belts of northern Kerala where intensity of aggregation is more severe than in south Kerala where arrival of summer rains (KDAF 2005; Vijayakumar et al. 2000) and subsequent beetle invasion is delayed six weeks, revealed that all field stages of *L. tristis* get six weeks more feeding time. Hence, the following sequence of events could possibly lead to comparatively very heavy increase of *L. tristis* in North Kerala: late onset of summer rains, entry of all larval and pupal stages into the adult stage from the absence of rainfall-mediated mortality, and the longer feeding time available for teneral adults of different age classes leading to greater consumption of reserve food materials by all age classes and increased survival during dormancy.

The total absence of larvae/pupae and the very low presence of adults in the field after the arrival of summer showers during both study periods indicate that rainfall is the most important factor driving them to dormancy and making it a very crucial event determining the life cycle of *L. tristis* in the region. The sudden disappearance and invasion into buildings following summer showers is not surprising as tenebrionids generally prefer dry habitats (Endrödy-Younga and Tschinkel 1993; Watt 1992) and present records shows that *L. tristis* is not an exception. However, it is not understood why they do not return to the field after the occasional showers in the first week of April/last week of March, which last for 5–10 days. Following summer showers, the leaf litter is dry for approximatly 45 days before the South West monsoon rains occur in June and the invading beetles could have returned to field instead of remaining in dormancy. We suggest that wetness “turns on” the irreversible physiological mechanisms to enter into dormancy (Mansingh 1971; Tauber and Tauber 1976; Denlinger 1986, 2002; Danks 1987; Leather et al. 1995) or probably, the perfect synchronism of *L. tristis* with rubber tree phenology/weather arose by means of natural selection.
